# The Computational Properties of a Simplified Cortical Column Model

**DOI:** 10.1371/journal.pcbi.1005045

**Published:** 2016-09-12

**Authors:** Nicholas Cain, Ramakrishnan Iyer, Christof Koch, Stefan Mihalas

**Affiliations:** Allen Institute for Brain Science, Seattle, Washington, United States of America; Indiana University, UNITED STATES

## Abstract

The mammalian neocortex has a repetitious, laminar structure and performs functions integral to higher cognitive processes, including sensory perception, memory, and coordinated motor output. What computations does this circuitry subserve that link these unique structural elements to their function? Potjans and Diesmann (2014) parameterized a four-layer, two cell type (i.e. excitatory and inhibitory) model of a cortical column with homogeneous populations and cell type dependent connection probabilities. We implement a version of their model using a displacement integro-partial differential equation (DiPDE) population density model. This approach, exact in the limit of large homogeneous populations, provides a fast numerical method to solve equations describing the full probability density distribution of neuronal membrane potentials. It lends itself to quickly analyzing the mean response properties of population-scale firing rate dynamics. We use this strategy to examine the input-output relationship of the Potjans and Diesmann cortical column model to understand its computational properties. When inputs are constrained to jointly and equally target excitatory and inhibitory neurons, we find a large linear regime where the effect of a multi-layer input signal can be reduced to a linear combination of component signals. One of these, a simple subtractive operation, can act as an error signal passed between hierarchical processing stages.

## Author Summary

What computations do existing biophysically-plausible models of cortex perform on their inputs, and how do these computations relate to theories of cortical processing? We begin with a computational model of cortical tissue and seek to understand its input/output transformations. Our approach limits confirmation bias, and differs from a more constructionist approach of starting with a computational theory and then creating a model that can implement its necessary features. We here choose a population-level modeling technique that does not sacrifice accuracy, as it well-approximates the mean firing-rate of a population of leaky integrate-and-fire neurons. We extend this approach to simulate recurrently coupled neural populations, and characterize the computational properties of the Potjans and Diesmann cortical column model. We find that this model is capable of computing linear operations and naturally generates a subtraction operation implicated in theories of predictive coding. Although our quantitative findings are restricted to this particular model, we demonstrate that these conclusions are not highly sensitive to the model parameterization.

## Introduction

For more than a century, neuroscientists have worked to refine descriptions of cortical anatomy, either differentiating or consolidating models of cortical circuits [1]. The notion that a fundamental neuronal circuit performs a canonical computation in neocortex, that can be generalized across species and areas, is of fundamental value to both experimental and theoretical neuroscientists. Douglas and Martin provided evidence for such a canonical microcircuit in the cat striate cortex, as well as a descriptive model of its structure [2, 3].

The fundamental building block of circuits on this scale is the cell type specific population. For example, the Douglas and Martin microcircuit model implicated distinct cell types and cortical laminae in its function. Each individual population in the circuit might perform linear or nonlinear transformations on its inputs, depending on the parameterization of the model [4]. The individual cells that make up the population might be spatially segregated (i.e. distinguished by layer) or might be intermingled, and distinguished by genetically defined cell type or projection pattern. The microcircuit can then be conceptualized as a modular collection of populations, with scale and composition dependent on function. Whole brain regions are assembled from ensembles of microcircuits that together perform its overall function, the clearest example being orientation columns in V1. Over time, the microcircuit model can be refined, constrained by including cell type specific parameterizations, synaptic properties, detailed microcircuit anatomy, and other relevant experimentally measured data of a particular cortical area.

When taken together, the cumulative result of multiple recurrently connected canonical circuits might perform the complex nonlinear computations necessary to implement models of higher-order cognitive function. Furthermore, many theoretical models of cortical processing involve hierarchical arrangements of processing stages, and the evidence for such a hierarchical organization, particularly in the visual system, is generally accepted (for example, [5]). Informed by the seminal work of Hubel and Wiesel [6] in the perception of orientation, the catalogue of algorithms for which there exists models relying on a staged, hierarchical implementation has grown significantly. Beyond perception, hierarchical theories include invariant object recognition (for example [7]; see [8] for a review), selective visual attention (see [9] for a review) and models of Bayesian inference via predictive coding (for example [10]).

In order to perform any of these hierarchical computations, individual elements within the hierarchy must perform an intermediate stage of processing. It is hypothesized that these intermediate stages implement a local canonical computation, and their hierarchical arrangement subserves (or even defines) a global information processing stream. In this study, we examine the computational properties of the Potjans and Diesmann [11] cortical column. The main focus of that study was the construction of a realistic computational model of cortex. Here we ask what type of computation this model might subserve as a candidate canonical model of cortical processing. Based on its properties, we then speculate about the role of such a processing unit in an abstract hierarchical computational scheme.

We find that simultaneous excitation to L2/3 and L4 offset in their effects on L5, in essence performing a subtractive computation between two step inputs. Additionally, we find that the model possesses a linear computational regime under the condition that incoming inputs do not preferentially target inhibitory or excitatory populations within a layer. We then examine the response of the model to sinusoidal inputs, again finding evidence of linear computation. In the discussion, we relate these findings to the role of such a processing element in light of theories of hierarchical computation.

## Materials and Methods

### 0.1 Model Parameterization

The Potjans and Diesmann [11] cortical column model is composed of 8 recurrently connected homogeneous populations of neurons, totaling approximately 80,000 neurons and .3 billion synapses. Each neuron receives background Poisson input, and is recurrently connected to neurons in other populations via a population-specific connection probability matrix derived by combining data and methods from several studies. In our study both network and single neuron parameters from [11] are used.

The population statistic approach used in Iyer et al. [12] assumes synapses that instantaneously perturb the voltage distribution of the postsynaptic population. Therefore, we assume that the fast kinetics of synapses in the Potjans and Diesmann cortical column (*τ*_*s*_ = .5 ms) can be well-approximated by the DiPDE formalism (For a discussion regarding the effect of non-instantaneous synapses, see [12], “Methods: Non-instantaneous synapses”). As a consequence, the coupling of these shot-noise synapses is instantaneous, perturbing the voltage distribution directly by a constant .175 mV for excitatory synapses, and -.7 mV for inhibitory synapses. These values are computed from the total charge resulting from a single synapse of weight *w* in [11] using their notation (See [12] for additional details):

$$\Delta v=\frac{Q}{C_{m}}=\frac{1}{C_{m}}\int_{0}^{\infty }I\left(t\right)dt=\frac{w}{C_{m}}\int_{0}^{\infty }\text{exp}\left(-t/\tau_{s}\right)dt.$$ (1)

Connection probabilities, synaptic weight distributions, and delay distributions are taken directly from [11] (with the exception of the L4e → L2/3e connection probability, which was doubled to .088, following [13]). This was done to define equal synaptic strength for all excitatory connections (The original strength for this one connection was doubled relative to other projections), while maintaining roughly the same overall projection strength. The connection probability was multiplied by the size of the presynaptic population to parameterize an effective multiplier (in-degree) on the incoming firing rate from a presynaptic population. Because they minimally impact the firing rate dynamics of the leaky integrate-and-fire model, refractory periods were simplified from 2 ms to zero.

**Fig 1:**
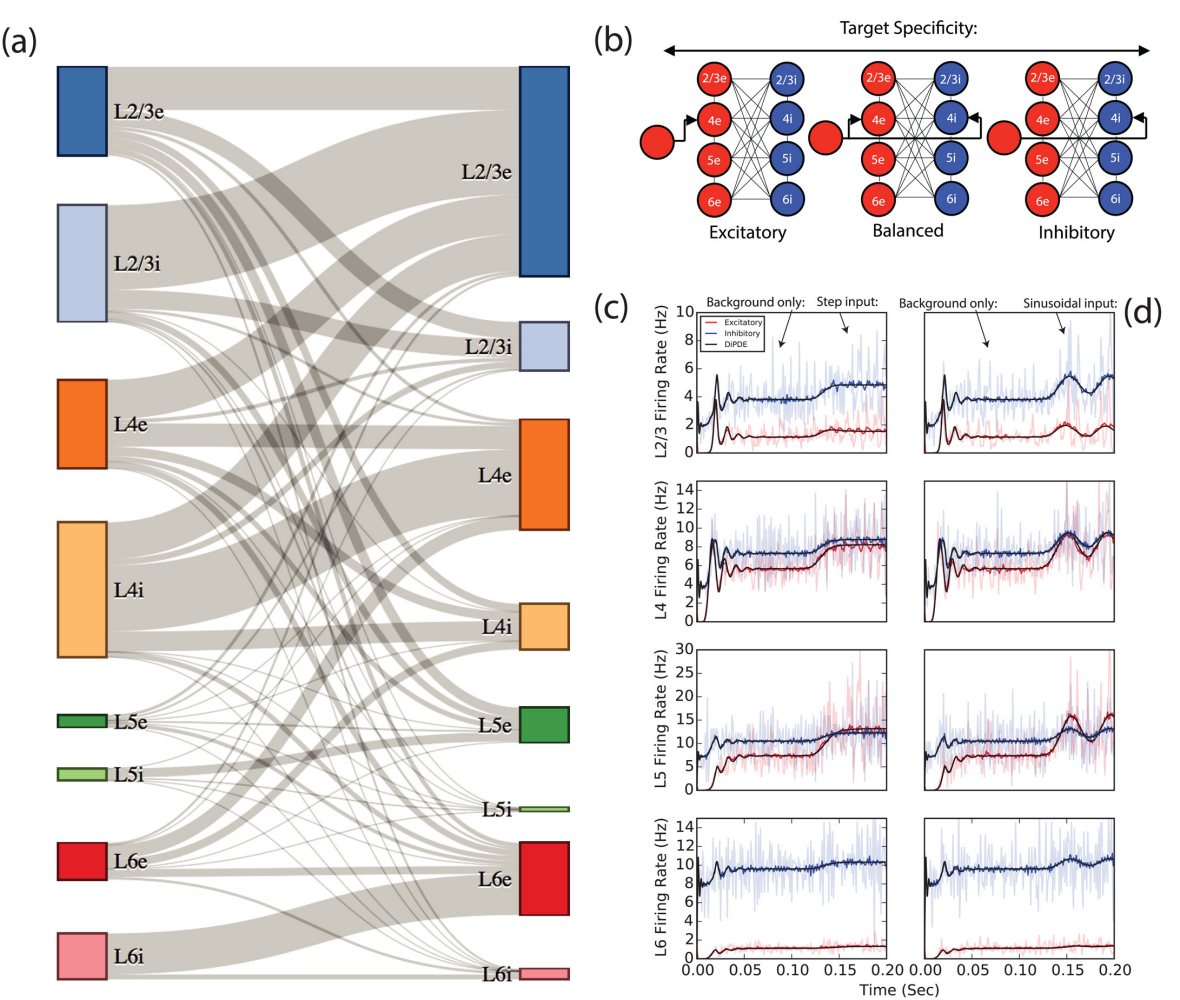
Model overview and comparison of population statistic model (DiPDE) with leaky integrate-and-fire (LIF) simulations. (a) Schematic overview of the connections between source (left) and target (right) populations. Line thickness corresponds to the number of projections between populations. The number of projections from inhibitory populations is scaled by 4 (the relative difference between inhibitory and excitatory synaptic strengths), so that excitatory and inhibitory projections can be visually compared. (b) The column model is perturbed in one of three ways: Excitatory, Balanced, or Inhibitory, illustrated here driving Layer 4. (c-d) Mean firing rate across all populations (1 ms bin width) of 100 averaged LIF simulations (solid fluctuating red trace for excitatory and blue for inhibitory subpopulation; a single example firing rate trace is rendered semi-transparent in the background) of the cortical column model. Black lines show prediction of DiPDE simulation across all layers under either (c) 20 Hz step or (d) sinusoidal with 20 Hz peak amplitude in firing rate, 24 Hz frequency inputs in excess of background excitation. At *t* = 100 ms, an additional input beyond the background excitation drives the excitatory population of layer 4 as illustrated in (b), “Excitatory”. Transient dynamics, steady-state firing rates, and responses to additional inputs are well approximated by DiPDE.

The only significant deviation from the Potjans and Diesmann model was a decrease in the mean background firing rate across all populations by a factor of 8.54, and subsequent increase in the synapse strength of these connections by an equal amount. This modification leaves the mean synaptic input from background unchanged from the original model, but increases the variance of this stochastic input. After this change, the intrinsic oscillations of the original NEST model are significantly damped (but not completely eliminated; see Fig 1). The matched DiPDE model does not exhibit intrinsic oscillations, although in general population density models are capable of exhibiting this phenomenon [14].

Each population is initialized to a normal distribution of membrane voltages, with a mean at the reset potential and standard deviation of 5 mV. Before application of any additional input (i.e., step or sinusoidal drive), background excitation is applied to each population as specified in [11], and simulated for 100 ms to reach a pre-stimulus steady state. When driving the model, additional layer-specific excitatory stimulus is input into the target layers(s), and simulated for an additional 100 ms. For step inputs, the difference of the final steady-state less the pre-stimulus steady-state (i.e. after discarding the initial start-up transient dynamics) define the layer-specific firing rate output perturbation.

### 0.2 Numerical Methods

In this study, all simulations of the model were performed using a numerical simulation of the displacement partial integro-differential equation (DiPDE) modeling scheme proposed in [12] with a time-step of.1 ms. At this temporal resolution, a 200 ms DiPDE simulation requires 31 seconds running on a 2.80 GHz Intel Xeon CPU. The corresponding NEST simulations [15, 16] included in Fig 1(c) and 1(d) require 402 seconds each (single processor), and results from 100 of these simulations are averaged to obtain the mean firing rate pictured. In each of these 100 averaged NEST simulations, connectivity matrices and initial values for voltages were randomized. The population density approach in computational neuroscience seeks to understand the statistical evolution of a large population of homogeneous neurons. Beginning with the work of Knight and Sirovich [17] (See also [18, 19]), the approach typically formulates a partial integro-differential equation for the evolution of the voltage probability distribution receiving synaptic activity, and under the influence of neural dynamics. Neuronal dynamics typically follow from the assumption of a leaky integrate-and-fire model. We implement a numerical scheme for computing the time evolution of the master equation for populations of leaky integrate-and-fire neurons with shot-noise current-based synapses (For a similar approach, see [20]).

$$\tau_{m}\frac{dv}{dt}=-v+\Delta v\sum_{i}\delta \left(t-t_{i}\right)\;\;\;\;\;\;\;\;\;\;v>v_{th}\Rightarrow v\to v_{r}$$ (2)

Here *τ*_*m*_ is the membrane time constant, *v* is the membrane voltage, Δ*v* is the synaptic weight, *v*_*th*_ is the threshold potential, and *v*_*r*_ is the reset potential (here taken to be zero for simplicity). Extending [12], each population receives input from both background Poisson input and recurrent connections from each cortical subpopulation. We emphasize that this is not a stochastic simulation; for example, the background Poisson drive is not a realization of a Poisson process, but rather the effect of a Poisson-like jump process on the evolution master equation. At each time step, a density distribution representing the probability distribution of membrane voltages for each population is updated according the differential form of the continuity equation for probability mass flux *J*(*t*, *v*) (Here *p*(*t*, *v*) is the probability distribution across *v* at time *t* on (−∞, *v*_*th*_); see [21] for more information):

$$\frac{\partial p}{\partial t}=-\frac{\partial J}{\partial v}$$ (3)

**Fig 2:**
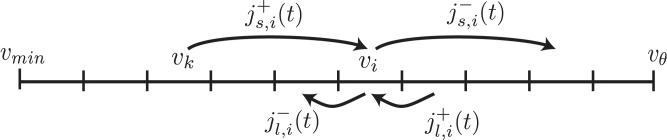
Illustration of probability mass flux. Assuming a single excitatory input with weight *w* = *v*_*i*_ − *v*_*k*_, at time *t* subdomain *v*_*i*_ has two sources $j_{\left(s,i\right)}^{+}\left(t\right)$ and $j_{\left(l,i\right)}^{+}\left(t\right)$ of probability mass influx, and two outflow destinations $j_{\left(s,i\right)}^{-}\left(t\right)$ and $j_{\left(l,i\right)}^{-}\left(t\right)$. As Δ*t* → 0, these fluxes are linear in the probabilities at each node *p*_*i*_ (Eqs 9–12).

The voltage distribution is modeled as a discrete set of finite domains (See Fig 2). Synaptic activation of input connections drive the flux of probability mass between nodes, while obeying the principle of conservation of probability mass. As a result, a numerical finite volume method is an ideal candidate for computing the time evolution of the voltage density distribution, and we numerically solve Eq 3 with a finite volume method.

The spatial (voltage) domain

$$D=[v_{min},v_{\theta }]\subset R$$ (4)

is subdivided into a set of non-overlapping subdomains

$$V=\{v_{i}\subset D\}.$$ (5)

Each subdomain contains a control node *p*_*i*_ that tracks the inflow and outflow of probability mass due to synaptic activation and passive leak. At each time step, *p*_*i*_ is updated by considering probability mass flow resulting from synaptic activation from all presynaptic inputs as well as leak; for simplicity we will describe the update rule assuming a single presynaptic input. Additionally, we will assume a single synaptic weight, although in general this approach works equally well for a distribution of synaptic weights. Under these assumptions, the discretized version of Eq 3 can be formulated as:

$$\frac{dp_{i}}{dt}=-\frac{\Delta J_{i}}{\Delta v_{i}}$$ (6)

$$\Delta J_{i}=f_{i+\frac{1}{2}}-f_{i-\frac{1}{2}}$$ (7)

$$=\left(j_{\left(s,i\right)}^{-}-j_{\left(l,i\right)}^{+}\right)-\left(j_{\left(s,i\right)}^{+}-j_{\left(l,i\right)}^{-}\right).$$ (8)

Here $f_{i\pm \frac{1}{2}}$ denotes flux across the right or left subdomain boundary, *j*_*s*_ denotes flux resulting from the input population (via synaptic activation), and *j*_*l*_ denotes flux from the leak; the superscript is a convenience that denotes the overall sign (i.e. inflow or outflow) of the contribution of the term to *p*_*i*_.

Synaptic activation contributes *j*_(*s*)_ to the overall flux by displacing probability mass (*p*Δ*v*) with a transition rate *λ*_*in*_, the presynaptic firing rate. By directly computing the probability mass flux as Δ*t* → 0 over the subdomain boundary (while enforcing probability mass conservation), the contribution of passive leak *j*_(*l*)_ to the overall flux can be formulated as a transition rate that increases exponentially with time constant *τ*_*m*_ as the voltage of the subdomain boundary being crossed increases. To summarize, the flux contributions to the *i*^*th*^ subdomain are:

$$j_{\left(s,i\right)}^{+}=p_{k}\Delta v_{k}\lambda_{in}$$ (9)

$$j_{\left(s,i\right)}^{-}=p_{i}\Delta v_{i}\lambda_{in}$$ (10)

$$j_{\left(l,i\right)}^{+}=\frac{p_{i+1}v_{i+\frac{1}{2}}}{\tau_{m}}$$ (11)

$$j_{\left(l,i\right)}^{-}=\frac{p_{i}v_{i-\frac{1}{2}}}{\tau_{m}}$$ (12)

Here the synaptic influx $j_{\left(s,i\right)}^{+}$ depends on *p*_*k*_, the probability mass in subdomain *v*_*k*_ located a distance *w* = *v*_*i*_ − *v*_*k*_ (the synaptic weight) from *v*_*i*_. In the special case of *i* = 0 and *w* > 0, the node that acts as the reset value for probability mass that exceeds the spiking threshold *v*_*θ*_ (i.e. the boundary condition) receives probability mass from all nodes less than *w* from *v*_*θ*_. Because these updates result from a linear update from probabilities, the entire time evolution can be formally represented as:

$$\frac{dp}{dt}=\left(L+S\right)p$$ (13)

where leak and synaptic input contributions have been separated into two separate discrete flux operator matrices. At this step, it is trivial to include additional synaptic inputs *S*_0_, *S*_1_, …*S*_*m*_, yielding a formal solution over a single time step Δ*t*:

$$p\left(t+\Delta t\right)=\text{exp}\left(\Delta t\left(L+\sum_{s=0}^{m}S_{s}\right)\right)p\left(t\right)$$ (14)

for some initial probability distribution **p**(*t*). At each time step, the synaptic input matrices *S*_*k*_ are updated to reflect the changes in firing rate of the presynaptic populations (if necessary).

Probability mass that is absorbed at threshold and inserted at the reset potential defines the fraction of the population that spiked; after normalization by the discrete time step Δ*t*, this defines the output firing rate. The output firing rate provides the rate of a Poisson process that drives any recurrently-connected postsynaptic populations. Probability mass flux through the boundary *v*_*θ*_ into the subdomain at *i* = 0 defines the instantaneous firing rate of the population, computed as:

$$\lambda_{out}\left(t\right)=\frac{\sum_{s=0}^{m}j_{\left(s,0\right)}^{+}}{\Delta t}$$ (15)

Recurrent coupling between simulated populations is accomplished by assigning *λ*_*out*_ of the presynaptic population to *λ*_*in*_ of the postsynaptic population.

The source code for DiPDE is released as an open source python package under the GNU General Public License, Version 3 (GPLv3), and is available for download at http://alleninstitute.github.io/dipde/. The package includes an example implementation of the cortical column model analyzed in the main text, absent any inputs in excess of background excitation.

## Results

### Step Inputs: Target Specificity and Laminar Computation

In this section we describe the repertoire of computations caused by step inputs over and beyond background excitation (See Fig 1(c) and 1(d) for an example simulation, compared to 100 averaged leaky integrate-and-fire (LIF) simulations) into a coarse-grained population-statistical version of the Potjans and Diesmann cortical column model (See Fig 1(a) for a visual summary of projections in the column model; for a complete model description see [11], Tables 4 and 5). The targeting of cell types (i.e. target specificity) has important consequences for the responses caused by incoming inputs. We examine the consequences of three types of target specificity, summarized in Fig 1(b) for incoming excitatory projections into a given layer within the column. The excitatory and inhibitory target specificity regimes excite their respective cell types, while the balanced regime does not preferentially target either subpopulation. Unless otherwise specified, the step input has a firing rate of 20 Hz, and models a convergent connection with 100 independent presynaptic sources per target neuron.

**Fig 3:**
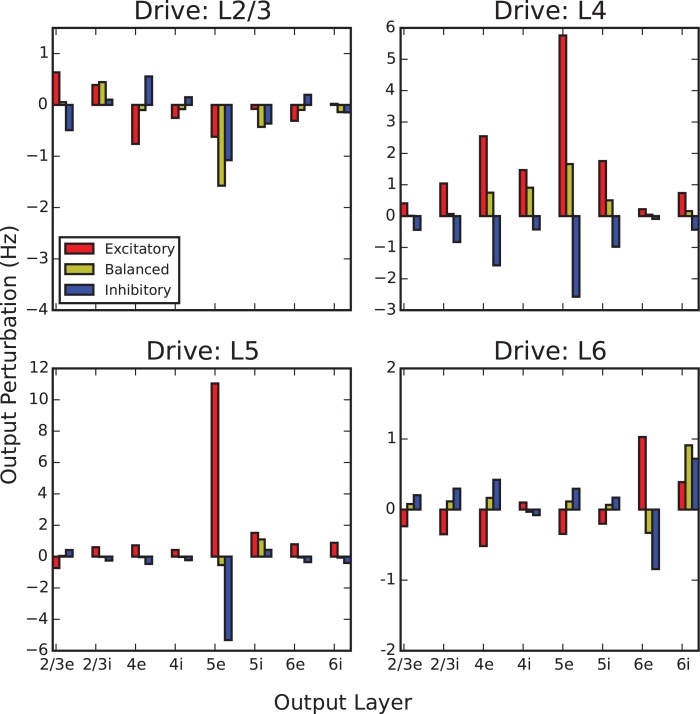
Summary of output perturbations evoked by single-layer step inputs. Step inputs are projected into a drive layer (L2/3, L4, L5, L6) under 3 different target specificity conditions. In all cases a 20 Hz firing rate input in excess of background excitation is applied at 100 ms, and output perturbation is defined as the difference between the eventual perturbed steady-state (200 ms) and the steady-state before perturbation. For example, balanced L2/3 drive (yellow, top-left panel) evokes an approximately 1.5 Hz decrease in firing rate in the L5e subpopulation, while balanced L4 drive (yellow, top-right panel) evokes a 1.5 Hz increase.

Fig 3 provides an overview of output perturbations evoked by step input into a given layer, under each target specificity condition. In effect, this provides an at-a-glance summary of the catalogue of computations that the cortical column can perform, given a 20 Hz step pulse excitatory input into a single layer.

**Fig 4:**
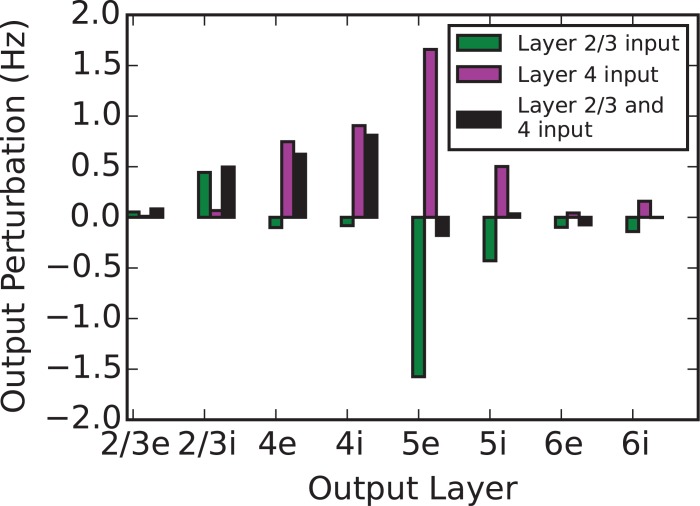
Perturbations resulting from balanced target specificity (equal excitatory and inhibitory input), L2/3, L4, and both together. The eventual change in steady-state of each subpopulation in the model differs in response to three balanced inputs. We focus on the response of the L5e and L5i subpopulations; the effect of inputs into L2/3, as compared to L4, are opposite and nearly equal. When both inputs are simultaneously applied, they approximately offset each other in their combined effect on the perturbation in L5. This indicates that L5 activity is representing the difference between the L4 and the L2/3 inputs.

We find that the effect of driving L2/3 under any specificity condition has a depressing effect on the activity in L5. In contrast, when driving L4 or L5, activity across almost every population in the network increases or decreases when driving the excitatory or inhibitory subpopulation, respectively. Fig 3 also demonstrates that under balanced target specificity (yellow), the effects of inputs into L2/3 and L4 are nearly equal-and-opposite with respect to the output of L5. We summarize this comparison across all output layers in Fig 4 which additionally plots the combined effect of inputs simultaneously into L4 and L2/3. This plot demonstrates that these two inputs approximately offset; we explore this observation further in the next section.

We note that the input layers involved in this subtraction are implicated in bottom-up vs. top-down comparisons in the theory of hierarchical predictive coding [22]. Also conspicuous is the output population reporting this subtraction; L5 pyramidal neurons provide the dominant cortical output, including the pons, striatum, superior colliculus, and to value encoding dopaminergic neurons in the VTA or SNc [23] where subtraction errors might skew reward expectations (see Discussion for further details).

### Step Inputs: Linear vs. Nonlinear Computation

**Fig 5:**
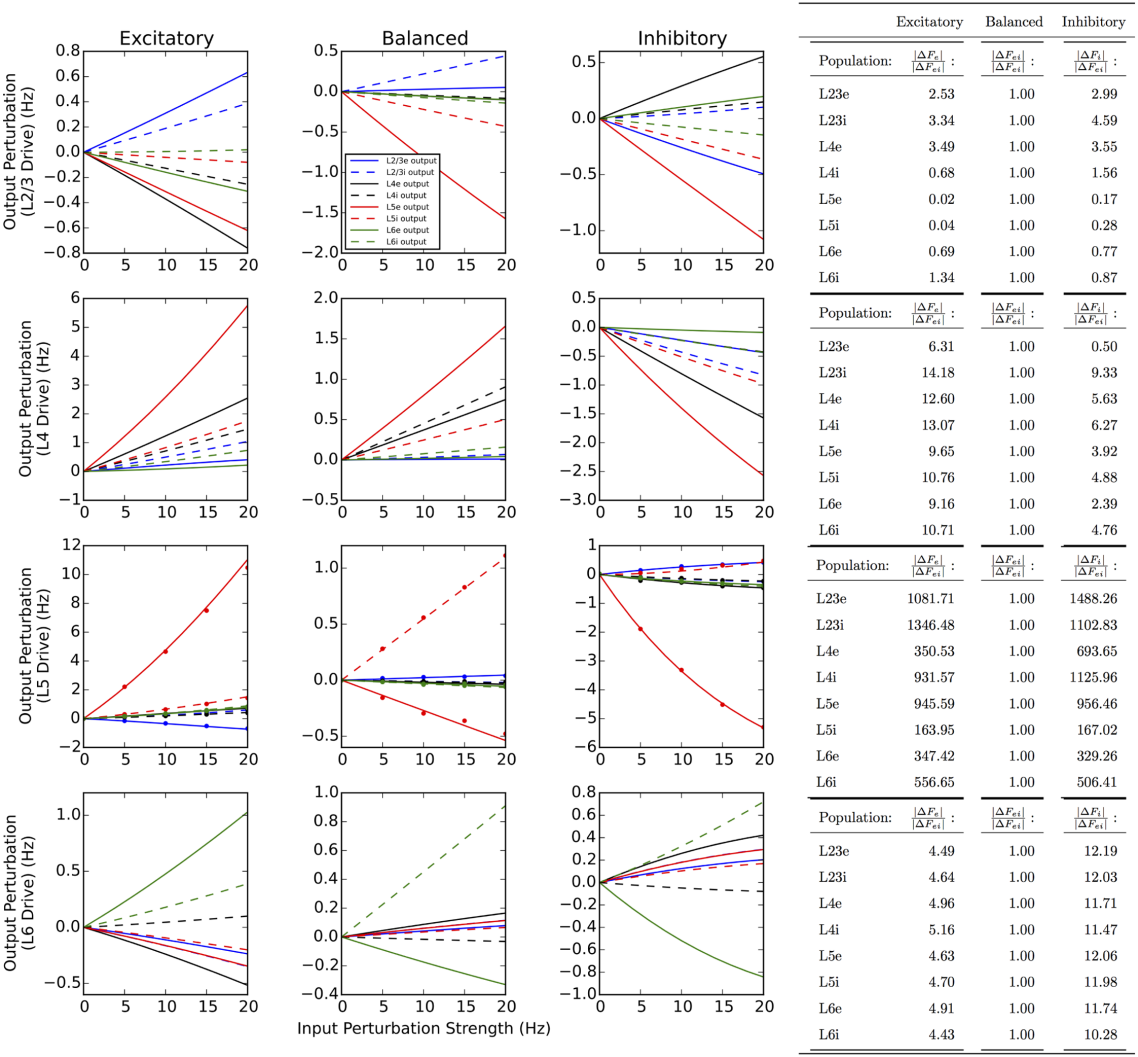
Homogeneity of output perturbations. As the magnitude of the input drive increases, the output perturbation responds either linearly (balanced target specificity) or nonlinearly, demonstrating that the target specificity of the incoming input shapes the linearity of the response. Solid and dashed lines indicate perturbations of excitatory and inhibitory populations, respectively (L2/3, blue; L4, black; L5, red; L6, green). For example, when L5 (i.e. 3rd row) is driven with balanced target specificity (middle column), the perturbations increase proportionally as input strength increases from 0 to 20 Hz. In contrast, when the inhibitory population is selectively driven (right column), the perturbation increases sub-linearly as input strength increases. Also included in the third row (dots) are results from 100 averaged leaky integrate-and-fire simulations (performed in NEST, see Methods), demonstrating that the trends observed in the population density simulations are present in the original system as well. To quantify linearity, the perturbation resulting from a 5 Hz amplitude step input is extrapolated to 10 Hz, and the percentage deviation of this prediction from the true perturbation relative to pre stimulus steady-state is computed. This relative error (See Main Text, Eq 16) is then normalized by the relative error from the balanced case, demonstrating that in general, balanced inputs result in more linear responses. The table on the right summarizes the relative errors for the different conditions.

Linear computations are characterized by simultaneously exhibiting homogeneity (i.e. multiplicative scaling in the sense of a linear map) and additivity with respect to inputs. The previous section examined output perturbations across layers and target specificity profiles of a single strength (20 Hz firing rate). In this section, we first examine the effect of linearly increasing the strength of the input, testing the homogeneity of the system. Fig 5 extends Fig 3 by providing a summary across an increasing range of input strengths.

Under balanced target specificity (middle column of panels), the magnitude of each population response exhibits a scaling behavior, linear in the input magnitude. In contrast, when neurons are targeted with a cell type specific bias, the response of certain subpopulations can be nonlinear. The clearest example of the nonlinear influence of the inhibitory subpopulation occurs when driving layer 5. Through both an increase in direct self-inhibition, and indirect reduction of self-excitation via the L5e subpopulation, excitatory drive into L5i can paradoxically decrease activity, an effect described previously in inhibition-stabilized recurrent networks [24]. Eventually this effect reverses when the L5e activity is completely inhibited.

**Fig 6:**
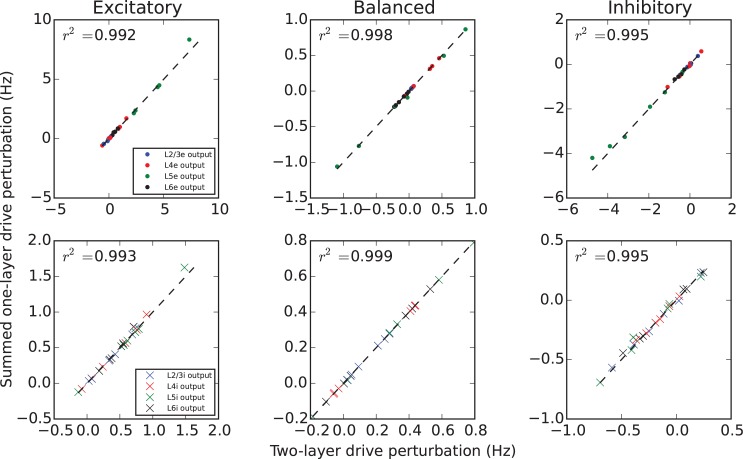
Additivity of output perturbations. All possible pairs of two input layers are selected to receive a 10 Hz step input in excess of background excitation. Dots and crosses indicate perturbations of excitatory (top) and inhibitory (bottom) populations computed by DiPDE, respectively (L2/3, blue; L4, black; L5, red; L6, green). For each possible pair of these input layers, the output of all populations is plotted versus the linear superposition of the same inputs taken independently, in two separate simulations (see main text for more details). Linear additivity of inputs between input layers results in points along the dashed identity line; this is the case for excitatory and balanced target specificity. As quantified by the coefficient of determination (*r*^2^) resulting from a linear fit, the balanced input regime results in a linear response, and deviates weakly from linearity when inputs are not balanced (See S2 Fig).

Fig 6 demonstrates that, likewise, additivity is violated (somewhat, as the points deviate from the identity line) when preferentially targeting inhibitory neurons. Each point in the figure depicts the result of driving two separate layers with a 20 Hz firing rate input, and considering the perturbation in firing rate of each subpopulation (specified in the legend). For a given target specificity condition, two independent simulations are run, for each of the two input layers; the sum of the perturbation they evoke is plotted on the vertical axis. The output resulting from a single simulation with two equal inputs into each input layer, is plotted on the horizontal axis. When a point lies along the identity line, this implies additivity.

This figure implies a conclusion similar to the homogeneity study above: as the target specificity moves from excitatory to inhibitory, the firing rate computation performed on laminar inputs by the cortical column changes from linear to weakly nonlinear. In the previous section, we demonstrated that balanced 20 Hz firing rate inputs to L2/3 and L4 approximately offset each other in the output evoked in L5. The homogeneity and additivity demonstrated above indicate that L5 will actually reflect a subtraction operation on these two inputs. We return to this point in the discussion.

### Sensitivity Analysis

Given the amount of recurrent connectivity in the model, its linear response to step inputs under balanced target specificity might seem surprising. However, it is known that balanced networks can exhibit linear responses to external inputs (See, for example, [25]). Although the model parameterization is taken from the literature, we also investigated the sensitivity of this linear response to perturbations in model parameters. By perturbing the connection probability matrix (Table 5 “Connectivity” in [11]), we defined 1000 alternative models. Specifically, each entry in the matrix was multiplied by a normally distributed random number with unit mean, and standard deviation taken as 5% of the entry (negative values were thresholded to zero).

The homogeneity of response to each new model was assessed by linearly extrapolating the perturbation resulting from a 10 Hz firing rate step input from the results obtained from a 5 Hz step input. The absolute value of the prediction error:

$$\Delta F=\left(F_{10}-F_{0}\right)-2\cdot \left(F_{5}-F_{0}\right)$$ (16)

quantifies the difference between the extrapolated value, and the true value obtained by direct simulation of a 10 Hz firing rate input. Here *F* indicates the firing rate after reaching steady-state, and the subscript indicates the strength of the step input. Intuitively, this quantity will be zero when a linear extrapolation can predict the data (i.e. a linear relationship between inputs and outputs). Nonzero values indicate the failure of a linear extrapolation, and thus a nonlinear dependence of the output firing rate on the input over the regime of 0–10 Hz perturbations.

S1 Fig shows a stacked histogram of this prediction error for the 1000 perturbed models, across all combinations of target specificity, laminar drive, and output population. Under balanced target specificity (middle column), the prediction error is reliably smaller, particularly when layers 4 and 5 are targeted (middle two rows). This implies that the linear relationship between inputs and outputs under balanced input of the original cortical column model is insensitive to small perturbations in the connection probability matrix.

A similar result holds for additivity predictions, shown in S2 Fig. For the same perturbed models, the additive prediction error is defined as the sum of output responses in two layers from two different simulations, minus the output resulting from driving the two layers in the same simulation. Again, the model under balanced target specificity is less sensitive to perturbations than when cell types are selectively driven. Therefore, we conclude that the observation of linear responses in model output in the previous section is not a result of fine tuned parameters.

### Sinusoidal Inputs: Linear Filtering

In the previous section, we demonstrated that target specificity can determine the linearity of the model response under step inputs. To further investigate the linearity of the transformation that the column applies on its inputs, we next consider sinusoidal drive above and beyond background drive (See Fig 1(d) for an example simulation, compared to 100 averaged LIF simulations). S3 Fig summarizes the nonlinear distortion in each populations response under a 5 Hz peak amplitude sinusoidal drive. Only responses with a peak amplitude greater than .05 Hz are plotted. Total harmonic distortion (THD) compares the power present in the harmonics of the driving frequency in the sinusoidal input signal that perturbs a subpopulation above and beyond the background firing rate:

$$THD\left(f\right)=\frac{\sqrt{\sum_{i=2}^{\infty }V_{i}^{2}}}{V_{1}}$$ (17)

**Fig 7:**
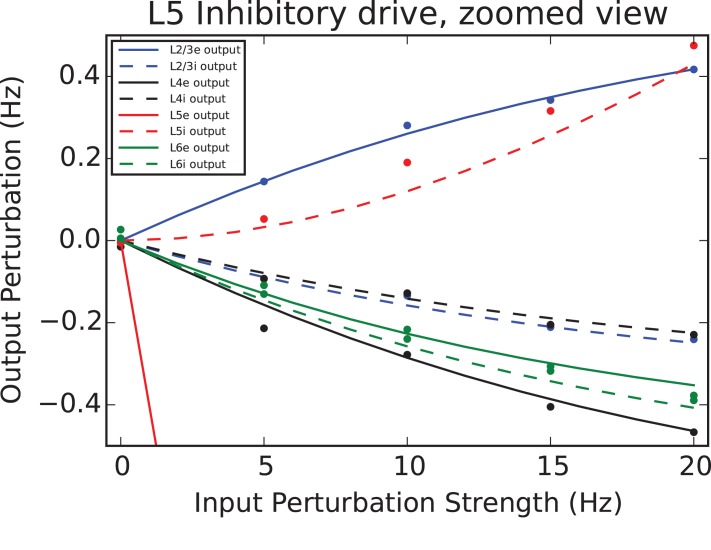
Nonlinearity resulting from drive into L5 inhibitory population. This expanded view of row 3, right column of Fig 5 demonstrates the strongly inhomogeneous response resulting from driving the L5 inhibitory population. Solid and dashed lines indicate perturbations of excitatory and inhibitory populations, respectively (L2/3, blue; L4, black; L5, red; L6, green). Simulations in NEST (100 averaged leaky integrate-and-fire simulations) are plotted as points.

Here *V*_*i*_ is the power spectral density (PSD, [26]) of the *i*^*th*^ harmonic of the principal (driving) frequency. This figure reinforces the conclusion from the previous section, that the target specificity of the sinusoidal drive can affect the nonlinearity of transformations resulting from population-level processing. In particular, balanced drive minimizes the harmonic distortion imposed by the dynamics within the model. In contrast, inhibitory drive into layer 5 produces nonlinear responses throughout the column, in agreement with observations about the homogeneity of responses to step inputs (cf. Fig 7)

The low THD of the output signals from balanced drive indicate that the firing rate *y*(*t*) of a population in the column model can be approximately modeled as a linear filter on the input signal *x*(*t*) plus a baseline *x*_0_:

$$y\left(t\right)=x_{0}+\int_{0}^{\infty }x\left(t-\tau \right)h\left(\tau \right)d\tau$$ (18)

**Fig 8:**
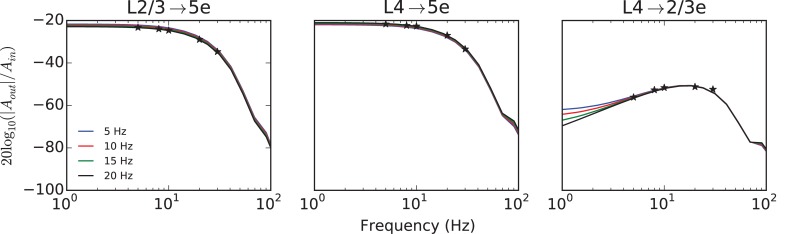
Amplitude attenuation of balanced sinusoidal drive. This figure summarizes the amplitude attenuation of 3 example transformations. Sinusoidal input with amplitude *A*_*in*_ drives superficial layers (L2/3 or L4), and expresses output in the L5e or L2/3e subpopulation with an attenuated amplitude *A*_*out*_. These filters are numerically computed at 4 different input amplitude strengths, and then normalized, further demonstrating linearity. The output from L5e (left and middle column) is lowpass filtered with a cutoff frequency near 15 Hz, corresponding to an RC circuit with a time constant near the membrane time constant (10 ms) of neurons in the system. The response of L2/3e in response to L4 drive is bandpass in the 10–30 Hz range. The averaged power spectral densities of 100 matched NEST simulations run at driving frequencies 5,8,10,20, and 30 Hz (20 Hz input amplitude) are plotted for comparison.

Fig 8 provides a numerically computed description of three examples of this linear filtering, resulting from balanced drive from L2/3→L5e, L4→L5e, and L4→L23e. Of all possible input/output pairs, these examples show the least signal attenuation from the amplitude *A*_*in*_ of the input signal *x*(*t*) to the amplitude *A*_*out*_ of the output signal *y*(*t*) (i.e. the largest impact on changes to subpopulation firing rate). Clearly evident in the first two figures are first-order lowpass filters, similar to feedforward systems found in [4] with significantly higher synaptic weights (relative to threshold). These filters both have a cutoff frequency near 15 Hz, implying a corresponding RC time constant near the membrane time constant (10 ms) of neurons in the system. Interestingly, this observation is in agreement with the very general prediction of predictive coding theories, that high frequencies should be attenuated when passing from superficial to deep layers [22] (See Discussion). Transmission from L4 to L2/3 is band-passed near the 10–30 Hz range.

## Discussion

In this study, we examine what input/output transformations a popular model of a cortical column performs on layer-specific excitatory inputs. Transformations are defined as perturbations to the steady-state mean firing rate activity of subpopulations of cells in response to step and sinusoidal inputs in excess of background drive. Because the mean firing rate is a population-level quantity, we use a population statistic modeling approach, by numerically computing the population voltage density using DiPDE (http://alleninstitute.github.io/dipde/), a coupled population density equation simulator (See Numerical Methods). This approach enables a fast, deterministic exploration of the stimulus space and model parameterization. Our approach begins with a data-driven model as a starting point, and then examines the computations its dynamics subserve, as opposed to fitting a model to a preselected set of dynamical interactions resulting from assumptions about cortical computation. Our goal is to discover robust evidence for theories of cortical function using knowledge about structure (synthesized by Potjans and Diesmann [11] into their cortical column model), while limiting biases and *a priori* functional assumptions.

We consider three discrete regimes of input specificity: excitatory preference, no preference (i.e. balanced, in which both excitatory and inhibitory cells in any one layer receive the same external input), or inhibitory preference. We find that balanced target specificity results in output perturbations that scale linearly with input strength and combine linearly across input layers. In contrast, selective targeting of a particular cell type (especially the inhibitory subpopulation) leads to nonlinear interactions. Additionally, we find that equal, simultaneous, and balanced inputs into L2/3 and L4 are offset in their effect on the L5 firing rate; combining this with the observation of linearity implies that perturbations in L5 activity represent a subtraction from L4 activity of L2/3 activity. The inhibitory effect of L2/3 input on L5e output appears to be largely mediated by L2/3 interneurons inhibiting L5 pyramids (c.f. [27], their Fig 4) while the excitatory effect of L4 on L5 is a network effect resulting from multiple projection pathways. We conclude that the cortical column model implements a subtractive mechanism that compares two input streams and expresses any differences in the mean activity of L5. While this computation can be implemented via other inputs, this combination is interesting because no target cell type specificity is required.

How does this observation of a mechanism for subtraction relate to existing theories of cortical processing? Predictive coding [10, 28] postulates a computation that compares an internal model of the external environment to incoming sensory signals, in order to infer their probable causes [29, 30]. The subtractive dynamics supported by the cortical column model could accomplish this. However, this would imply that sensory signals are represented dynamically in one layer, an environmental model in the supragranular layer, and that their functionally relevant difference is relayed by the infragranular layer (layer 5). The internal granular layer (layer 4) is the obvious candidate for incoming environmental evidence, given its specialized role in receiving input from the primary sensory thalamus. Similarly, the role of the infragranular layer in driving subcortical structures involved in action (basal ganglia, colliculus, ventral spinal cord) seem compatible with the proposition of layer 5 representing the output of a comparison operation. Although more speculative, this leaves the supragranular layer responsible for generating the internal environmental model, which seems reasonable given its abundance of intracortical projections and increased development in higher mammals.

These speculative roles of the various cortical layers conform to abstract models of canonical microcircuits (See, for example, [31]). This is especially true when placed in a hierarchy of processing stages, for example in hierarchical predictive coding (hPC) [22]. In this framework, sequential processing stages generate top-down predictions, and pass bottom-up prediction errors, at each level in the hierarchy. In primates, the laminar segregation of these streams is easily aligned with the anatomical characterization from Felleman and Van Essen [5], with feedforward connections targeting L4, and feedback connections avoiding L4. In rodents, the relation between lamination and hierarchy is less clear [32]. Although the central theme of distinct populations of forward-projecting neurons targeting L4 vs. backward projecting neurons avoiding L4 in the visual system seems conserved [33], these distinct populations are not segregated by layer, but instead intermingled [34]. Therefore, future experimental attempts to establish connections between hierarchically defined visual processing regions and theoretical models may require projection-target-segregated (or perhaps genetically-segregated, if projection markers can be established), as opposed to laminae-segregated, cellular subpopulations.

An additional connection between the hPC model and the results of the simulations in this study is presented in Fig 8. Here the response of the deep layer of the model to stimulation in either of the two superficial layers is well characterized by a linear low-pass filter. Interestingly, this filtering is a prediction of hPC, where high frequencies should be attenuated when passing from superficial to deep pyramidal cells [22]. The low-pass filtering prediction arises from the hypothesis that cortex is performing a form of Bayesian filtering, by attempting to update an estimated quantity using noisy measurements. These noisy estimates by their nature have higher-frequency content than the uncorrupted “true” quantity being estimated, and so the appearance of a smoothing transform is not surprising. However, it is surprising that our model, formulated without assuming any underlying computation (especially not Bayesian filtering), performs this smoothing at a dynamical stage precisely where the anatomically-informed hPC model requires it. Taken together, the convergence of experimental, anatomical, theoretical, and simulation evidence is striking.

As mentioned above, neurons in the infragranular layer project both cortically and subcortically. Based on the hPC model, and because of the model’s ability to compute a subtraction between inputs to the granular and supragranular layers, we have speculated this computation could represent an error signal between reality and expectation. What would this conclusion imply for the subcortical projections? Watabe-Uchida et al. [23] found that dopaminergic neurons in the ventral tegmental area and substantia nigra pars compacta receive sparse input from the deep layers of cortex (for example their Fig 5). Because of the well established role of these midbrain structures in valuation, motivation, and reinforcement learning, these authors suggest that these dopaminergic neurons might “calculate the difference between the expected and actual reward (i.e., reward prediction errors).” While speculative, it is possible that this prediction is calculated cortically and relayed either directly or indirectly [35].

There are a number of concrete steps that can be taken to strengthen the relationships between model, theory, and experiment. A more comprehensive model parameterized by experimental data with additional layers and cell types could be combined with matched optogenetic *in vivo* and *in silico* perturbation experiments. These manipulations could validate model predictions, suggest refinements, and test specific conclusions related to theories of population-based cortical processing, for example the functional role of different classes of genetically defined interneuron populations. Such models might also suggest a reinterpretation of how different cell populations contribute to the computation of error signals, or suggest new canonical computations carried out by population-level activities. Either way, in our view, the population density modeling approach will continue to provide a valuable tool for quickly exploring the dynamical consequences of population level computational models.

## Supporting Information

S1 Fig Homogeneity of output responses is insensitive to changes in connection probability when target specificity is balanced. Stacked histograms demonstrate the effect of randomly perturbing the connection probability matrix on linear prediction error (See Eq 16). Entries in the connection probability matrix between populations in the cortical column model were randomly perturbed (See Main Text, Sensitivity Analysis). The results of 1000 perturbed networks are used to construct a stacked histogram (8*1000 total cases), with the abscissa indicating the difference between prediction of the 10 Hz response extrapolated from the 5 Hz response and observed firing rate at 10 Hz input (similar to Main Text, Fig 5). The value of the unperturbed model is indicated by markers along the abscissa, at the top of each panel. In general, there is a wider histogram (higher variance), i.e. higher sensitivity to connection probability perturbations, when target specificity is not balanced (first and third columns), especially when L5 is driven (3rd row). This is indicative of a stable operating point in model space (w.r.t connection probability matrices) for linear computations, under balanced drive.
(EPS)

S2 Fig Additivity of input responses is insensitive to changes in connection probability when target specificity is balanced. Stacked histograms (8*1000 total cases) of the additivity prediction error (i.e. abscissa less ordinate from Main Text, Fig 6) across all possible layer pairs indicate that balanced inputs (10 Hz) into multiple layers can be linearly composed. The value of the unperturbed model with respect to this measurement is indicated by markers along the abscissa, at the top of each panel. When inputs are not balanced (first and third columns), nonlinear interactions cause output perturbations that are not a superposition of individual inputs, indicated by a mean that is not centered around zero. Additionally, the magnitude of this effect is sensitive to the connection probabilities of the model indicated by the larger variance.
(EPS)

S3 Fig Total Harmonic Distortion as a function of frequency. For all layers, and across the three target specificity conditions, total harmonic distortion (THD, Eq 17) is plotted versus the driving frequency of the sinusoidal input (15 Hz Poisson spike rate peak-to-peak amplitude). In general the distortion of sinusoidal drive is low.
(EPS)
